# Causal Explanation from a Conceptual Engineering Perspective: Non-Reductionism, Precision, and Pluralism

**DOI:** 10.1007/s10441-026-09538-3

**Published:** 2026-09-06

**Authors:** Jaehyun Lee, Lauren N. Ross

**Affiliations:** Department of Logic and Philosophy of Science, University of California, Irvine, Irvine, USA

**Keywords:** explanation, conceptual engineering, philosophy of biology, philosophy of neuroscience

## Abstract

While projects in philosophy of science often focus on similar sorts of topics, there are often massive differences in how philosophers approach them. One approach that has enjoyed significant success—and that is modeled in the work of Wimsatt and others—involves taking a conceptual engineering perspective in analyzing these topics. In this paper, we outline main features of this conceptual engineering approach and use it to advance three emerging claims regarding causal explanation in biology and neuroscience. We suggest that causal explanation in these domains is *non-reductive* in contrast to always requiring (or improving with) lower-level details, that it is guided by *precise* explanatory targets in contrast to complete or multidimensional explanatory targets, and that it is *pluralist* in involving importantly distinct types of causal systems.

## Introduction

Many projects in philosophy of science focus on similar types of foundational topics, concepts, and questions. These topics are often central to scientific inquiry, such as causation, explanation, representation, confirmation, (anti)realism, and reductionism, among many others. While projects in this literature share a focus on similar sorts of issue, there are often massive differences in how philosophers approach them. One approach that has enjoyed significant success—and is modeled in the work of Wimsatt—involves taking a means-end and functional perspective in analyzing these topics (Wimsatt 2007). In this approach, philosophers are likened to “cognitive engineers” or “conceptual engineers,” as they evaluate scientific concepts, practices, and methods in terms of the means, functions, and goals they serve (Wimsatt 2007; Woodward 2021). Advantages of this approach are its ability to capture successes of science, to specify the rationale behind forms of scientific reasoning, and to provide guidance on challenging issues. This distinguishes this approach from others that merely (or mainly) describe scientific practice without capturing its principled nature. As Wimsatt states, “an adequate philosophy of science should have normative force” and “mere descriptions of scientific practice ... will not do” (Wimsatt 2007, 26). The normative guidance available with this approach stems from specifying scientific goals and assessing whether our theories of these topics “work” toward and well serve these goals. Other key features of this approach are its attention to scientific practice, methods, and challenges, but also to limitations that scientists face and strategies that they use.

A classic example of this conceptual engineering approach is found in Wimsatt’s work, while other examples and discussions are present in the literature (Wimsatt 2007; Woodward 2021).^1^,^2^ In this paper, we outline main features of this conceptual engineering approach and use it to advance three emerging claims regarding causal explanation in biology and neuroscience. In particular, we suggest that causal explanation in these domains is (a) *non-reductive* in contrast to always requiring (or improving with) lower-level details, that it is (b) guided by *precise* explanatory targets in contrast to complete or multidimensional explanatory targets, and (c) that it is *pluralist* in involving importantly distinct types of causal system. We aim to address these points in ways that engage with the challenges that scientists face, that capture the “order” in their practice (Wimsatt 2007), and that are useful for scientific theorizing.

## A Conceptual Engineering Approach

This paper examines and builds on a conceptual engineering approach to philosophy of science as modeled by Wimsatt, Woodward, and others (Wimsatt 2007; Woodward 2021). We do this by focusing on the topics of causal explanation, primarily in biology and neuroscience. Hallmarks of this conceptual engineering framework include careful attention to the methods, strategies, and concepts found in scientific practice, with an eye toward the goals and functions they serve. As Wimsatt states, this perspective views “scientific processes ... as end-directed or teleological activities, generating a conceptual engineering of science, with natural resonance with design analyses in engineering” (Wimsatt 2007, 5). On this approach, it is suggested that many scientific practices, methods, and concepts have evolved and been shaped in ways that ensure that they function for various goals. When clearly specified, these goals can serve as benchmarks for assessing whether such practices work, are justified, and are successful. If not met, such goals can supply reasons and guiding standards for revising scientific practices, methods, and concepts.

In the work of Wimsatt (2007) and Woodward (2021), this conceptual engineering approach is often characterized as involving two main standards—a descriptive standard and a normative standard. The descriptive standard ensures that philosophical accounts engage with and capture actual scientific practice. In considering philosophical accounts of scientific explanation, this standard expects such accounts to be descriptively accurate in reflecting explanations that scientists provide, in a way that helps us understand their logic and structure. This requires appreciating the types of phenomena scientists seek to explain, the challenges they face, the limits placed on their information and decision-making, and the goals they expect their explanations to serve. As Wimsatt states, this approach aims to capture what “real scientists” are up to, the methods we “actually have” in scientific work, and a “realistic” picture of science (Wimsatt 2007, 5). In this manner, it focuses on science “in practice,” in contrast to science “in principle” (Wimsatt 2007). On this view, philosophy of science should have sufficient fidelity to and engagement with scientific work. Producing philosophical work that meets this standard is no small task—it requires that philosophers gain significant proficiency in the complexities of scientific language, practice, and methodology. However, while this standard is critical, it isn’t the only important benchmark to meet. If philosophical work *only* meets this descriptive standard, this can be viewed as mere “science reporting” in detailing merely what scientists *do*—how scientists carry out their work, but not much more (Woodward 2023, 16). If scientists disagree about how explanations work, or what explains some target of interest, philosophical research that is only descriptive is unable to offer guidance and solutions (it would just report the conflict). Ideally, we want to capture actual practice, but also the underlying principles that guide successful scientific work and that can offer guidance when new complexities, challenges, and conflicts arise.

This is where our second normative standard helps, which is characteristic of this two-part framework. Suppose our focus is on the topic of scientific explanation. We do not want our philosophical accounts of explanation to just capture how scientists *do* provide explanations, but also how they *should* provide them. Now, in our view, scientists often do provide explanations that are successful, but this isn’t always the case as seen in situations where researchers disagree (and they can’t all be right). Specifying how scientists should engage in these projects relates to clarifying how scientists are successful relative to various goals of interest. For example, if explanations are expected to provide information relevant to the goals of control, prediction, and making generalizable inferences, we can assess candidate explanations according to how well (or poorly) they serve these goals. This allows us to argue that, if scientists are interested in these particular goals, then they should accept one explanation over another, on the basis of the fact that it better serves their goals. Inherent to this approach is the view that, if methods, descriptions, and concepts are found in scientific practice, we should have a default assumption that they serve some function, and we should take efforts to understand what this is. If scientists distinguish types of causal systems—causes that are deterministic versus probabilistic, proximal versus distal, and strong versus weak—we should look for the function of these distinctions. Our philosophical work should also meet more than just this normative standard. It isn’t suitable for our philosophy to be prescriptive, but removed from scientific practice. If work only meets this normative standard, it can be criticized as “armchair” philosophy, that is informed by mere intuitions but devoid of engagement with science. This “armchair” philosophy can misrepresent actual challenges scientists face, it can fail to capture phenomena they want to explain and reason about, and it can focus on toy examples that poorly capture the phenomena they study.

We rely on this conceptual engineering approach to argue for three main claims in the context of causal explanation in biology and neuroscience. We suggest that causal explanation in these domains is: (a) non-reductive in contrast to always requiring (or improving with) lower-level details, that it is (b) guided by precise explanatory targets, and that it is (c) pluralist in involving importantly distinct types of causal systems.

## Explanation as Non-Reductive

Interest in reduction spans diverse topics in the philosophical literature (Hüttemann and Love 2011; Wimsatt 1976; Bickle 2006). Here, we focus on explanatory reduction claims, which emphasize the importance of lower-level causal detail in explaining higher-level outcomes. We suggest that non-reductive forms of causal explanation are common, principled, and justified in biology and neuroscience, and that explanatory practice does not always fit the strict reductive picture that is sometimes advocated in the literature.

Consider two main explanatory reduction claims in the philosophical literature. One claim suggests that, given some explanatory target of interest (1) the details that explain this target are always at a lower-scale or level than the target itself (Machamer et al. 2000; Craver and Bechtel 2007; Bickle 2006). Examples of this claim are present in mechanistic accounts of explanation, which suggest that “lower level entities, properties, and activities are components in mechanisms that produce higher level phenomena” (Machamer et al. 2000, 13).^3^ These accounts emphasize the “hierarchical” nature of mechanisms and the sense in which some phenomenon is explained “by describing its *underlying* mechanism” (Craver 2007, 108, emphasis added). These views are related to purported limitations on causation—endorsed by various new mechanists and other researchers—such as claims that causal relationships are limited to “levels” in ways that rule out the possibility of downward causation and interlevel causation. As Craver and Bechtel state, “the idea of causation would have to stretch to the breaking point to accommodate interlevel causes” and “there are no causal interactions beyond those at a level” (Craver and Bechtel 2007, 547, 561). Similarly, new mechanists state that scientists identify explanatorily relevant causes by drilling down and “decomposing” systems into their lower-level parts (Bechtel and Richardson 2010), which conflicts with a picture in which explanatorily relevant causes are at higher-levels than the explanandum itself. Thus, the search for explanatorily relevant causes on these views involves looking for causes at lower-levels.

A second explanatory reduction claim in this literature cites a purported (2a) infinite regress situation, in which it is claimed that explanations can always (and perhaps should always) cite increasingly lower-levels of detail, as there are no principled reasons to stop (Railton 1981). Another version of this claim maintains that (2b) there are always details at lower-levels that can be included in explanations to improve explanatory power (Sober 1999). Examples of these claims are found in Sober’s discussion of reduction and the “explanatory capabilities of higher- and lower-level sciences” (Sober 1999, 544). Sober suggests that, when it comes to what makes an explanation “objectively” superior to another, the lower-level details do the “work” in producing higher-level outcomes (Sober 1999, 548). He maintains that the only disadvantage of including additional lower-level detail is that it “explains too much” and that, while we “may not want to hear the gory details ... that does not mean that the details are not explanatory” (Sober 1999, 549).^4^ Similarly he states that “physics in principle can explain any singular occurrence that a higher-level science is able to explain [although] the level of detail in such physical explanations may be more than many would want to hear, but a genuine explanation is provided nonetheless” (Sober 1999, 561). Other versions of these claims are found in the new mechanist literature, where it is suggested that mechanisms in a scientific domain “bottom out” with “fundamental” components that capture where “the explanation comes to an end” (Machamer et al. 2000, 13).^5^ Similarly, new mechanists often state that abstract, higher-level, and less detailed causal structures are ‘incomplete’ and reflect a ‘shallowness’ of understanding (Craver and Darden 2013).

While explanations in biology and neuroscience are sometimes reductive in the sense of (1), we suggest that this is not always the case and that many legitimate non-reductive explanations are found in these domains as well. An indication that (1) does not always hold—and that there is support for non-reductive forms of causal explanation—is that biologists and neuroscientists frequently cite higher-level causes in explaining lower-level outcomes. Examples are cases in which stressful life events are causally implicated in explanations of major depression (Kendler et al. 1998), environmental factors causally contribute to normal development and disease outcomes (such as the causal influence of sunlight and fertilizer on plant height and the causal influence of diet and exercise on diabetes), social factors like racism influence cortisol levels (Richman and Jonassaint 2008), and social structures causally influence health outcomes (where these structures include public policies, food deserts, etc.) (Haslanger 2016; Ross 2024b). These cases suggest that, as a descriptive matter, scientific practice in these domains does involve explaining lower-level outcomes by appealing to higher-level causes. These examples also reveal that scientists do not adopt the purported limitations on causation that new mechanists assume. This suggests that a non-reductive picture of explanation meets a descriptive standard in capturing aspects of actual scientific practice. These cases provide descriptive support for scientists who cite higher-level causes in explaining lower-level outcomes.

In addition to meeting a descriptive standard, these non-reductive explanations also meet a normative standard in serving various scientific goals. A main goal of causal explanation is control—in particular, identifying causes that “make a difference to” and provide information about control over some explanatory target of interest. In the examples above, the higher-level causes that scientists highlight meet this standard—these examples can be understood with an interventionist account, in which if a candidate cause were (hypothetically) manipulated, in some background circumstances, this would produce changes in some effect (Woodward 2005). For example, evidence suggests that manipulating stressful life events—making them more or less present—is causally relevant to and provides control over depression outcomes. A similar rationale holds for other examples that involve reference to proximal-distal causation (Ross and Kendler 2026), downward causation (Noble 2016), and discussions of higher-level constraints that limit effects at lower scales (Silberstein 2021; Raja and Anderson 2021; Ross 2024a, 2023). In these cases, higher level causes are useful in offering control, predictions, and generalizable inferences for lower scale outcomes. We do not suggest that there is a single higher-level cause that offers a full or complete explanation. Instead, we argue that these higher level causes are explanatorily relevant in these cases, their explanatory role is not mysterious, and they well serve various goals of interest. This captures a form of non-reductive causal explanation that has both descriptive and normative support.

This analysis also helps engage with the strong reductive claims in (2a) and (2b). We agree that biologists and neuroscientists are often attracted to a reductive picture in which it is important to appreciate details at increasingly lower-levels. However, these scientists also rarely view all of these lower-level details as required for giving explanations, but they can also struggle to say why this is the case. This is where a conceptual engineering approach can help. Consider explanatory targets such as neural firing, fruit fly eye color, and measles. In explaining each of these targets, scientists emphasize particular causes without citing details down to the lower-levels of fundamental physics. Scientists explain neural firing by citing the role of excitatory neurotransmitters, ions, ion channels, and synaptic vesicles on downstream firing. Fruit fly eye color is explained by the influences of genes, enzymes, and metabolites on eye development. Measles is explained by exposure to a virus that impacts immune system components and downstream physiological systems. Scientists select, highlight, and emphasize particular causes as having explanatory power—their explanations stop at the level these causes reside at, in contrast to going to increasingly lower and lower levels of detail from physics. One way to capture why scientists stop at these levels is that the causes at these levels (in contrast to fundamental physics) better serve the goals of control, prediction, and supporting inferences that generalize when it comes to the explanatory target.^6^ Once an explanatory target is fixed, the level of causal detail that matters is the level with causes that provide control over this target. If we are interested in explaining measles at the population-level, the measles virus provides the best control over this target—this has been demonstrated with vaccinations and there are no known lower-level causes that offer better population-wide control. In other words, current science has not identified factors at lower-scales that better explain, “make a difference to,” and control this population-wide disease. This captures the sense in which “explanation isn’t a game of how low can you go, but what gives you control” (Ross 2025b). Importantly, this claim does not deny that entities in lower-level physical science are *present* in these systems (of course they are), it denies that they are *explanatory* for the target of interest. In other words, the appropriate “aim of what are called reductionist explanations in science is not to atomize phenomena to the lowest possible level, but to be able to articulate and understand entities, events, and processes at diverse levels, and to give explanations involving heterogeneous relations among them in producing complex phenomena” (Wimsatt 2007, 4).

What about claims that future science will identify physics explanations for these examples? Here again, Wimsatt’s work and a conceptual engineering approach offer guidance. Our interest here is in actual scientific research—we are interested in science *in practice,* not science *in principle* (Wimsatt 2007, 9). These future-focused in principle claims are often contentious and reliant on guesswork, as there is often little consensus on the future that is envisioned. Our interest in this conceptual engineering approach is to capture the success, reasoning, and methods of our best current science without invoking guesses about what the future will hold.

## Explanation as Guided by Precise Explanatory Targets

Scientific explanations are often separated into an explanatory target, the *explanandum*, and factors that do the explanatory work, the *explanans*. Many accounts of scientific explanation present a standard picture in which scientists fix an explanatory target and then identify factors that explain, “account for,” and are “responsible” for this target (Hempel 1965, 247). The explanatory target (explanandum) is often expressed in terms of a why-question, while the explanation (explanans) is viewed as an answer to this question. Accounts of scientific explanation tend to pay significant attention to the explanans, which is what counts as explanatory once a target is chosen. However, much less attention is paid to the standards that well-defined explanatory targets need to meet and how challenging it can be for scientists to formulate suitable explanatory targets. The importance of well-defined explanatory targets is seen in examples where purported explananda remain unclear in problematic ways. For example, Nagel claims that the explanatory target and the why-question “Why do humans have lungs?” is too “ambiguous” to qualify as a well-defined explanatory target (Nagel 1961, 19). His rationale is that such a question is not sufficiently clear—it could be asking why humans evolved to have lungs or how lungs function physiologically (among other possibilities). Many other scientific concepts, terms, and labels run into a similar issue (due to lack of precision). Examples include attempts to explain consciousness, intelligence, “the mind,” and even disease outcomes (Kandel et al. 1994). Even for systems that are more clear and tractable, there are often highly distinct properties or features of the system that are of explanatory interest, while some concepts or questions can fail to specify which exact property is the target of explanatory inquiry.

We suggest that well-defined explananda need to be precise in ways that are not captured by many philosophical accounts. To see examples of these common views (which we find problematic), consider claims that explanatory targets should be sufficiently complete, full, and capture multidimensional aspects of a phenomenon (Craver and Bechtel 2007; Craver 2007). For example, Craver suggests that explanatory targets capture “multifaceted” phenomena and that proper explanatory targets should provide a “complete characterization of the phenomenon” of interest (Craver 2007, 126). Furthermore, Craver goes on to make separate but similar claims about the completeness of the explanans. In particular, he suggests that “good explanations” will account for all aspects of the phenomenon of interest (as opposed to just some aspects) (Craver and Bechtel 2007, 161). These points are seen in Craver’s discussion of mechanistic explanation:

First, one needs to add the core normative requirement that mechanisms must account *fully* for the explanandum phenomenon. Ideally, it is not enough to account for just normal input-output conditions. One must also account for the *multiple features of a phenomenon*, including its precipitating conditions, manifestations, inhibitory conditions, modulating conditions, nonstandard conditions, and byproducts. *Good explanations account for all of the features of a phenomenon* rather than a subset.” (Craver and Bechtel 2007, 161, emphasis added)

In other places, Craver highlights that the “central criterion of adequacy for a mechanistic explanation is that it should account for the multiple features of the phenomenon” (Craver 2007, 139). In our view, this is a problematic criterion for many reasons. For any phenomenon of interest, (i) it is possible to specify so many distinct features that listing them all is too extensive a project to be practical (or feasible), (ii) all of these features could never be captured in a single explanandum, and (iii) even if some large set of features for a phenomenon were collected into a single explanandum, it is hard to imagine how any single explanation (or explanans) could account for all of them. For example, suppose the final product of a metabolic pathway is our explanatory target and there are various features of this final product we are interested in. Among the features we might want to explain are why the product has: a particular production rate (versus another), a particular boiling point (versus another), a particular melting point (versus another), a particular density (versus another), a particular kinetic energy (versus another), a particular refractive index (versus another), a particular radioactive element composition (radioactivity) (versus another), a particular viscosity (versus another), a particular enantiomer composition (versus another), and the list goes on. Here we have various “aspects” of the phenomenon and their associated explanatory why-questions or explananda. While these are all features of the system, it does not make sense to combine all of these into a single “complete” explanatory target—they capture distinct explananda, which are explained by different details in these systems. Not only is it challenging to see how this type of “multifaceted” explanatory target could be captured in a framework for explanation, but such claims fail a descriptive standard as they are not supported by actual scientific practice. We agree with Nicholson (2012) that this multifaceted explanatory target standard “is problematic as actual scientific practice demonstrates that mechanistic explanations are never exhaustive catalogues of all the causal relations necessary for the production of phenomena” (Nicholson 2012, 159).

Instead, we suggest that well-defined explanatory targets should be precise in contrast to complete, full, or multifaceted. Explanatory targets should be precise in terms of specifying (a) particular properties of interest and also (b) the exact changes, contrasts, and variations in these properties that one wants to explain. Notice that above examples of explanatory targets such as consciousness, intelligence, and the mind can fail this first (a) standard. In these cases, a single scientific term can refer to different properties of explanatory interest. Unless it is clear which property is intended, the explanatory target can remain ambiguous. In these cases, scientists may talk past each other as they use the same terms (“consciousness”) but actually have different explananda (or phenomena of interest) in consideration. Examples that better meet standard (a) are explanatory targets such as fruit fly eye color, gene expression, neural firing, plant height, and measles. While each of these better specifies some *property* of interest, a further required feature for well-defined explanatory targets is (b) the specification of *changes* in this property that are of interest. Standard (b) helps prevent another type of ambiguity, where it isn’t clear what the contrast of the explanatory-why question (and explanandum) is. This ambiguity type is seen in Nagel’s worry in which asking “why do humans have lungs?” can be interpreted as distinct why-questions. This is resolved by clarifying the changes in the property or contrast of interest—for example, why human beings have lungs in contrast to evolving differently. Similarly, in explaining fruit fly eye color, it should be specified what differences in eye color types one wants to explain—red eye color versus white, red eye color versus black, or red eye color versus all other types. Different contrasts—even when they concern the same property (eye color, lungs, etc.)—can capture distinct explananda that require different explanations (explanans).^7^ We agree with Potochnik that “deciding on the precise explanandum, or how to characterize the event to be explained, helps determine” what qualifies as explanatory for this target (Potochnik 2020, 145).

Our approach suggests the importance of precise explanatory targets over complete, multifaceted explanatory targets. The points above highlight various normative strengths of precise explanatory targets. Essentially, identifying relevant causes (that meet the goals of control, prediction, and generalizable inferences) requires sufficiently clear specification of what exactly the target is. In other words, we cannot provide an explanation unless it is sufficiently clear exactly what we want to explain. Imprecision, lack of clarity, and ambiguity about the target stop this process in its tracks. This emphasis on precise explanatory targets also has descriptive support. This is seen in requirements for clear dependent and independent variables in experimental work (Shadish et al. 2002), attention to concept ambiguity in science (as with the “jingle fallacy” (Kelley 1927, 64)), and the absence of examples of “multifaceted” explanatory targets. This framework is compatible with accounts of explanation that are more “local” and precise, in contrast to “theory of everything” views that expect explanations to account for all or numerous features of systems (Wimsatt 2007; Woodward 2017). In this manner, our account agrees with Woodard in that it “requires that it be possible for a model or theory to explain some explananda having to do with some aspects of the behavior of a system without the model explaining all such aspects” (Woodward 2017). This is also compatible with the Wimsattian suggestion of “local rather than global order” and “a local kind of problem-solving” that captures clear (but perhaps also narrow) questions and answers, in contrast to broad, global, all encompassing explanations (Wimsatt 2007, 6).

## Explanation as Pluralist

Causal pluralism, as discussed in the philosophy of science literature, takes several forms. It can refer to distinct definitions of causation (definitional pluralism), different methods used to establish causation (methodological pluralism), and “distinctions within causation” (distinctional pluralism) (Woodward 2021; Ross 2025a). Debates about definitional pluralism concern what counts as the hallmark or characteristic features of causation, or the “primary” features of causation (Ross 2025a). These features are used to establish “whether a causal relationship exists” or not, in contrast to offering more “precise” information about the causal relationship (Griffiths and Tenenbaum 2005). Debates about definitional pluralism have received significant attention in philosophy, and different accounts of causation take different stances on what counts as the primary features of causation (Ross 2025a). Methodological pluralism about causation pertains to whether causation can be established (or identified) with one method or multiple distinct methods. While randomized controlled trials (RCTS) are viewed as a powerful method for establishing causation, there are debates about whether this is the only successful method, or if other methods can be used to establish (get sufficient evidence for) causation as well.^8^,^9^ Distinctional pluralism has received increasing attention in recent work, where there are efforts to systematically capture causal varieties that matter in life science domains (Woodward 2010; Ross and Woodward 2022; Ross 2021b). Where definitional pluralism concerns primary features of causation, distinctional pluralism concerns “secondary features” of causation—these are features that capture distinct types of causal influence, without being features that can be used to establish that causality is present (Ross 2025a). An example of this is Griffiths and Tenenbaum’s contrast between “causal structure” and “causal strength” in cognitive science research. Causal structure relates to “whether a causal relationship exists,” while causal strength pertains to “how strong that causal relationship might be” (Griffiths and Tenenbaum 2005, 334). This provides a helpful approach in considering further secondary features that commonly show up in the life sciences as it separates (i) whether a causal relationship is present or “exists” (primary features) from (ii) additional features that causal relationships can have, such as how strong, stable, and specific they are, and so on (secondary features).

Here, we focus on the third type of causal pluralism above, distinctional pluralism, and causal varieties that frequently arise in biology and neuroscience. Distinctional pluralism meets our descriptive standard in the sense that scientists reference causal varieties in their work. Life scientists use distinct terms to refer to different types of causes, causal relationships, and causal systems. This is seen in the use of complex causal concepts used to refer to complex causal systems, such as mechanisms (Woodward 2011; Wimsatt 2007), pathways (Ross 2021a; Boniolo and Campaner 2018; Bickle 2006), cascades (Ross 2018), circuits (Ross 2025b; Alon 2020), and so on. In other situations, biologists and neuroscientists refer to causes as “constraints” (Ross 2021c, 2023; Raja and Anderson 2021; Silberstein 2021), as “structures” (Ross 2024a), as deterministic or probabilistic, as reversible versus irreversible (Ross and Woodward 2022), and as differing in terms of specificity, strength, and stability (Woodward 2010, 2006). These terms seem to point to features that differ across genuine types of causal relationships and causal systems. This indicates, as Wimsatt suggests, a kind of “heterogeneous,” “tropical rainforest ontology” of causal structures in the world in contrast to a simple, homogeneous (Quinian) “desert ontology” (Wimsatt 2007, 6). In taking seriously our conceptual engineering approach, it is worth considering whether these concepts serve various functions in causal and explanatory reasoning. One reason to think that they do is that some of the secondary features that these terms pick out make a difference to how causal systems are studied and how they support goals such as prediction, control, and leading to inferences that generalize.

For one class of examples that supports distinctional pluralism, consider complex causal terms such as “mechanism,” “pathway,” and “cascade” (Wimsatt 1974; Ross 2025b). The mechanism concept has received huge amounts of attention in philosophy, biology, and neuroscience, often in connection to providing explanations (Craver 2007; Bechtel and Richardson 2010; Ross and Bassett 2024). As Wimsatt suggests, “[a]t least in biology, most scientists see their work as explaining types of phenomena by discovering mechanisms, rather than explaining theories by deriving them from or reducing them to other theories, and *this* is seen by them a reduction, or integrally tied to it" (Wimsatt 1974, 671). For Wimsatt and others, mechanisms are causal systems that are reductive, in the sense of having lower-level causal parts, that work together to produce some higher-level outcome of the system (Wimsatt 1974, 188). This reductive picture is related to the view that, in mechanisms, the “causally interrelated parts will be hierarchically organized” and that mechanisms are discovered through “decomposing” systems into their relevant lower-level parts (Bechtel and Richardson 2010). This reductive character of mechanisms is related to their association with machines, where paradigmatic machines (like watch mechanisms and car engines) also contain lower-level causes that interact to produce system-level behavior.

While mechanism is a common causal concept in biology and neuroscience, other causal concepts are also frequently used in these fields. Other causal concepts that are frequently found include pathways and cascades. Pathway examples include biochemical, cell signaling, gene expression, developmental, and vascular pathways (Ross 2021a). Examples of cascades include the blood coagulation cascade, cascading reactions in physics and chemistry, trophic cascades in ecology, and the cascading spread of infectious diseases (Ross 2018). While these causal systems need not contain a different definition (or account) of causation, they do appear to have features that distinguish them from mechanisms and other causal systems. While reductive, lower-level causes are characteristic of mechanisms, this is not seen in pathways and cascades, which appear to have their own unique features. Pathways often capture more higher-level causal routes, along which some entity flows—this is seen in the flow of blood along vascular pathways, signals along signaling pathways, an organism along developmental pathways, and metabolites along biochemical pathways. These causal systems capture higher-level causal routes and causal connections, where these often abstract from lower-level details (and mechanisms) that might instantiate the pathway. This is seen in the fact that scientists often analogize pathways to highways, freeways, and city streets, and represent scientific pathways with “pathway maps” of causal routes in some domain. Unlike mechanisms, these pathway maps provide information about how some entity can move through the connections. These maps provide information about what final destinations can be reached from a causal starting point, and which different starting points can lead to the same destination.

Cascades, alternatively, have the unique feature of sequential amplification, in which an initial small cause leads to increasingly larger effects. This is seen in illustrations of cascades in science journals and textbooks, as they often depict this one-to-many amplification feature. For an example of sequential amplification, consider enzyme cascades in which a sequence of enzymes is activated in succession (as seen in the blood coagulation cascade). To see this conceptually, consider a situation in which one unit of enzyme A produces ten units of enzyme B and one unit of enzyme B produces ten units of enzyme C. The activation of one unit of enzyme A in this system leads to a gain of 100, as the process produces 100 units of C for every unit of A. In enzymatic cascades, the gain at each step (and overall) is much greater than this example, leading to a massive amount of clotting factors in a short amount of time.^10^ Biological and neurological cascades often rely on amplification to serve various functions (such as signal transmission or quick and massive production, among others). However, they can also be present non-functionally, where they lead to damage as seen in ischemic cascades in neuroscience, trophic cascades in ecology, and disaster cascades in sociology. Additionally, various cascades are exploited by scientists in methods such as polymerase chain reaction (PCR) (Ross 2018). When scientists describe cascades, they often analogize them to the ripple effect, the snowball effect, and cascading waterfalls, because all of these systems also involve the sequential amplification feature. Unlike mechanisms, cascades do not always contain causal parts at lower-scales that influence higher-level outcomes. This is seen in enzyme cascades in which causes are same-level, and in energy cascades in which higher-level causes influence lower-level outcomes (Richardson 2007, 66; Wilson 2022).

A conceptual engineering approach suggests a departure from traditional, all encompassing “imperialist” views of mechanism, where mechanism it is said to capture all causal explanations in biology and neuroscience (Kaplan 2017). Although, some mechanist philosophers have claimed that “mechanism” can capture these other causal terms (and causal systems) (Craver 2007; Robins and Craver 2009), a normative standard suggests otherwise. This is because appreciating these distinct causal systems supports various scientific goals. While mechanisms are classically studied with “decomposition” and methods that “drill down,” pathways and cascades are not always amenable to such methods. This is because cascades sometimes have same-level or higher-level causes relative to their effects and pathways abstract from lower-level details. In fact, pathways are often studied with unique tracer and tagging methods in which some material is tagged and the tag is followed to reveal steps of the pathway. This is seen in radioactive tracer methods used to reveal metabolic pathways, ecological pathways, and vascular pathways—a technique that is not seen in standard mechanisms that lack the reliable flow of some entity along causal steps. Additionally, cascades explain explosive, powerful, and massive effects, which are not accounted for with causal mechanisms and pathways. This also captures why causal cascades are exploited in various technologies and how they can lead to massive destruction, damage, and collapse in various systems. Finally, similar to our earlier points about non-reductive explanation, both pathways and cascades abstract from significant lower-level details to highlight main, unique, and important causes that produce outcomes. While mechanisms often encourage acquiring more and more lower-level detail, pathways and cascades resist this heuristic. These distinct causal systems support a view of causal pluralism according to which distinctions among causes should be “helpful, illuminating, and useful in scientific contexts and to understand scientific reasoning" (Ross 2025a).

In our view, attention to scientific practice—its order and principles—supports a similar lesson for other causal varieties, such as causal constraints, causal structures, specific causes, and so on. Our default position should be to assume that scientists’ use of these causal distinctions plays a functional role in their work. We should take efforts to understand what this functional role might be, in efforts to capture the “order” in practice and a “theory of practice” that is present in the life sciences (Wimsatt 2007, 6, 9). This work supports a kind of principled pluralism about causal explanation in biology and neuroscience—a pluralism that should be accepted when it meets relevant descriptive and the normative standards. In sum, distinctional pluralism meets our descriptive standard in appreciating the diversity of causal concepts actually found in the life sciences. Additionally, it meets our normative standard in that these diverse causal concepts—and the systems they refer to—have distinct implications for causal investigative methods, level of explanatory detail, and types of outcomes these systems predict, control, and explain.

## Conclusion

This paper examines and develops a conceptual engineering approach with a focus on the topic of causal explanation in biology and neuroscience. We have outlined how the conceptual engineer is guided by descriptive and normative standards, and how this guidance can be applied to particular topics in the philosophy of science literature. As seen in this analysis, this approach emphasizes elements found in scientific practice and the function of these elements when it comes to concrete scientific goals. This approach appreciates that scientists are “limited beings,” that they study “messy systems in the real world," and that they gain traction—however piecemeal—in understanding the natural world (Wimsatt 1974). In continuation of this approach, we have applied its main standards in an effort to suggest three main points. In efforts to provide causal explanations in these fields, it is advantageous to (i) accept higher-level or abstract causal systems as explanatory without needing to include lower-level detail, to (ii) identify precise explanatory targets (in which the property of interest and changes to the property are clear), and to (iii) use frameworks that capture the diversity of causal systems, instead of shoehorning all systems into a single mechanistic framework. We have suggested that these principles are present in scientific work and also that they have a normative rationale. We intend for these points to be “useful to scientists” (Wimsatt 1974, 30)—perhaps for capturing obscure heuristics, helping settle debates, and crystallizing standards in their work—although we admit that this aim is nontrivial and requires engagement with the challenges, puzzles, and topics that scientists face in their work. In addition to helping advance the philosophical literature, such a conceptual engineering approach also has the significant benefit of showing the value of philosophy of science to various projects, such as scientific methodology, science communication, and assessments of various standards in science. These important influences of philosophy of science are modeling in the work of Wimsatt (1974) and numerous other philosophers, and we hope that they remain an aim in future work in our field.

## Data Availability

No datasets were generated or analysed during the current study.

## Footnotes

1 For further discussion and examples of conceptual engineering—and related notions such as Carnap’s “explication” and Haslanger’s “ameliorative analysis”—see: Carnap 1950; Haslanger 2012; Machery 2025; Dutilh Novaes 2020.

2 Even Carnap’s discussion of explication refers to an “engineering ideal” (Carus 2012, 225).

3 Here we follow the common practice of interpreting levels in terms of an intuitive, “layer-cake”, and scale (or size) picture, which is found in philosophy and science, but often remains underspecified. Such a notion is referenced in Sober’s mention of the “familiar hierarchical picture ... [that] ... runs (top to bottom) as follows—the social sciences, individual psychology, biology, chemistry, and physics” (Sober 1999, 543). While talk of levels is common, it is also difficult to clearly pin down and interpreted in different ways (Potochnik 2021).

4 These points are related to a form of “ruthless reductionism” that “construes lower levels as the source of all explanation” (Bechtel and Richardson 2010, xxxvii) (Bickle 2006).

5 For example, Machamer et al. state that “in molecular biology and molecular neurobiology, hierarchies of mechanisms bottom out in descriptions of the activities of macromolecules, smaller molecules, and ions” (Machamer et al. 2000, 14).

6 For another example of this, see the analysis of the “smoking explains lung cancer” example and multiple realizability (Ross ﻿^2020^).

7 A more colloquial example of problematic explanatory-why question ambiguity involves the famous bank robber Willie Sutton. After he was asked, “Why do you rob banks?” he responded with “Because that’s where the money is”. Of course, this reveals the interpretation of one contrast (why rob banks *versus* some other place) versus the intended one (why rob any place *versus* not robbing at all). While contrasts are often assumed and left unspecified, these examples reveal issues when multiple interpretations are likely.

8 The Journal for the American Medical Association (JAMA) restricts the use of causal language in submitted papers to studies in which randomized clinical trials were performed. As noted, for “all other study designs ... methods and results should be described in terms of association or correlation and should avoid cause-and-effect wording” (JAMA 2023).

9 Of course, the position one takes on the primary features of causation has implications for what methods will (and will not) establish causality.

10 This is similar to how a single patient with covid can transmit covid to 10 patients, and each of these secondary patients can each transmit covid to 10 patients—leading a single patient with the disease to produce a large amount of cases in the population.
